# Effectiveness of an Antenatal-Exercise Counseling Module on Knowledge and Self-Efficacy of Nurses in Northeast Peninsular Malaysia: A Quasi-Experimental Study

**DOI:** 10.21315/mjms2020.27.3.9

**Published:** 2020-06-30

**Authors:** Ismail Nor Azura, Ishak Azlina, Zakaria Rosnani, Mohd Noor Norhayati

**Affiliations:** Department of Family Medicine, School of Medical Sciences, Universiti Sains Malaysia, Kubang Kerian, Kelantan, Malaysia

**Keywords:** counseling, education, exercise, knowledge, nurses, pregnancy, self-efficacy

## Abstract

**Background:**

Because of their frequent, regular contact with antenatal mothers, healthcare providers play an important role in promoting the health benefits of antenatal exercise. This study aimed to determine the effectiveness of an antenatal-exercise counseling module on knowledge and self-efficacy of staff nurses.

**Methods:**

A quasi-experimental design was conducted. The intervention and control groups consisted of 66 nurses randomly selected from the Tumpat and Pasir Mas districts, respectively, in Kelantan. The intervention group received an antenatal-exercise counseling module, and the control group performed counseling based on self-reading. Knowledge and self-efficacy were assessed at the baseline and at week 4. Analysis of variance and repeated measure analysis of covariance were performed using SPSS.

**Results:**

There was a significant difference in the knowledge scores [estimated marginal mean (95% confidence interval, CI): 33.9 (33.29, 34.53) versus 27.4 (26.52, 28.29); *P* < 0.001)] and the self-efficacy scores [estimated marginal mean (95% CI): 31.3 (30.55, 32.03) versus 27.4 (26.03, 28.74); *P* = 0.005)] between intervention and control groups at week 4 after adjusting for duration of practice and formal training.

**Conclusion:**

The antenatal-exercise counseling module is recommended for use in routine counseling in health centers to promote healthy lifestyles among pregnant women.

## Introduction

Physical exercise is beneficial to the general population because it promotes good physical health. Exercise is believed to have similar benefits for expectant mothers and is believed to pose no risks to maternal or foetal health (1). Pregnant women are encouraged to engage in aerobic and muscle-strengthening exercises before, during and after pregnancy (2). Aerobic or endurance exercise involves movements of the large skeletal muscles, including those in the arms and legs, and includes swimming, brisk walking, cycling, and aerobic dancing. Pregnant women also benefit from exercises that strengthen the abdominal muscles, pelvic floor, buttock, and thigh muscles, which are effective in preventing and reducing backaches during pregnancy (3). Nevertheless, to accommodate anatomical and physiological changes in pregnant women, some modification of routine exercise activity is necessary.

A randomised controlled trial in Brazil showed that antenatal exercises contributed to lower gestational weight gain in overweight women (4). Antenatal exercise improves overall fitness, cardiovascular health, and muscle performance, lowers blood pressure, and protects against gestational diabetes mellitus. During labour and delivery, exercise helps to reduce the need for obstetric intervention or caesarean section (5). Physical exercise also eases the discomfort caused by physiological changes during pregnancy, including digestive problems, insomnia, anxiety and depression (1). No association has been found between the level of physical exercise and the intensity of low-back pain during pregnancy (6). Once pain develops, low-intensity exercise can reduce pain and significantly increase spinal flexibility (7).

Healthcare providers, particularly nurses, have frequent contact with antenatal mothers; therefore, they play an important role in promoting antenatal exercise. A survey of nurse midwives in the United States regarding their use of antenatal exercise counseling found that 65% of respondents provided individualised counseling regarding antenatal exercise (8). A study in Pennsylvania involving 24 pregnant women found that they characterised health advice on exercise and gestational weight gain as insufficient, often inappropriate, and therefore unlikely to influence them to engage in physical activity (9). Another study of community nurses and pharmacists found that they regularly provided guidance on nutrition and physical activity to pregnant women. Thus, continuous education is required to ensure that healthcare providers have appropriate knowledge (10).

In Malaysia, the Prenatal Care Manual is the main local reference for healthcare providers in handling antenatal mothers from pre-pregnancy up to puerperal period. This manual was made available for healthcare providers but not suitable for layman reference as it was developed for clinical purposes. It recognises antenatal exercise as one of the important components of antenatal care. It recommends that pregnant women without medical problems or complications participate in aerobic exercise and muscle-strengthening exercise (11). Apparently, healthcare providers do not comply with this guideline, possibly because of insufficient knowledge and inadequate training. Antenatal exercise module might be one of the options in enhancing their knowledge as well as improving their self-efficacy in terms of confidence level in delivering counselling. To date, there was no module on aerobic exercise for pregnant women available as a guide for the healthcare providers in giving counselling. The education material available in the country focuses more on muscle strengthening, breathing exercise and proper posture during pregnancy despite of great benefits of aerobic exercise.

Antenatal exercise has positive effects on both mother and foetus. Regular physical exercise helps overweight or obese pregnant women prevent excessive weight gain. In the rural Malaysian district of Kelantan, the prevalence of women who gain excessive weight during pregnancy is about 13% (12). A higher prevalence has been demonstrated in urban Malaysian populations: 29.4% (13). Considering the relatively high prevalence of excessive weight gain among pregnant Malaysian women, advising them to engage in physical exercise during pregnancy is appropriate. However, they are believed to receive minimal advices from healthcare providers pertaining to aerobic antenatal exercises. This is probably because of insufficient knowledge, inadequate training and lack of confidence among the healthcare providers in advising pregnant mother to exercise. The objective of the present study was to compare the knowledge and self-efficacy of staff nurses in the intervention group, who used the antenatal-exercise counseling module, to that of the nurses in the control group, both at baseline and at week 4. We hypothesised that the knowledge and self-efficacy scores would be higher in the intervention group than in the control group at week 4. Knowledge of counseling on antenatal exercise was assessed using a 36-item questionnaire. Self-efficacy on such counseling was defined as an individual’s belief in or level of confidence regarding delivering such counseling, and it was assessed using a 7-item questionnaire.

## Methods

### Study Design and Sample

The study used a community-based, quasi-experimental design and included, nurses and nurses-midwives working in the Maternal and Child Health Clinic, and who did not practice antenatal-exercise counseling. The study excluded community nurses and those involved in managerial work. Quasi-experimental design based on place of work was applied in this study. In quasi-experimental design, the different treatment conditions are not assigned to units at random because researchers may not have the choice possibly due to practical constraints (14). Two of 10 districts in Kelantan were selected due to its approximately similar healthcare service provision. Tumpat district has nine and Pasir Mas district has seven health clinics with maternal and child health services. Nurses from health centres in Pasir Mas were selected as the control group whereas nurses from Tumpat were selected as the intervention group. This was done to avoid interaction or contamination between the control and intervention groups.

The sample size was calculated based on comparing two means using PS software with an 80% power of study, a level of significance of 0.05 and a standard deviation of the total knowledge score of 3.7 (pilot study). After considering that the detectable difference in the total knowledge scores of the control group and the intervention group was 2, that the ratio of the control group to the intervention group was 1 and that the non-response rate was 20%, the calculated sample size for each group was 66 subjects. The inclusion criteria were nurses and nurses-midwives who were working in the Maternal and Child Health Clinic and did not practise antenatal exercise counselling prior to this study. Community nurses were excluded in this study.

### Research Tools

#### Antenatal-exercise counseling module

The module addresses the benefits of antenatal exercise, physiological changes during pregnancy, contra-indications for antenatal exercise and exercises that are safe and those that are not during pregnancy. The exercises that are safe are moderate exercise, which include brisk walking, static cycling and aerobic exercise. Moderate-intensity exercise is defined as having an energy requirement of 3 to 5 metabolic equivalents. In the absence of medical or obstetric complications, it is recommended that pregnant women exercise moderately for 30 min on most days of the week (15). The module was developed based on current guidelines, including (i) the Committee Opinion on Physical Activity and Exercise during Pregnancy and the Postpartum Period (2); (ii) the Malaysian Perinatal Care Manual (11); (iii) the Malaysian Dietary Guideline 2010 (16); and (iv) the Manual on Antenatal and Postnatal Exercise in Health Clinics (17). Information from these sources was used to create a table-top flipchart and a pamphlet that contained simple terms in the local language. This content was reviewed by an obstetrics and gynecology consultant, a family-medicine specialist, a sport-medicine specialist, and physiotherapy experts, in which it was revised and amended accordingly.

#### Questionnaire

Based on a literature review, the questionnaire was constructed to obtain sociodemographic data and responses on knowledge and self-efficacy. Knowledge on counseling regarding antenatal exercise was assessed using 36 items and responses were recorded as ‘true,’ ‘false’ or ‘not sure.’ For each ‘correct’ answer, a score of 1 was awarded. For each ‘wrong’ or ‘not sure’ answer, a score of 0 was awarded. The scores ranged from 0–36. Self-efficacy on antenatal-exercise counseling was assessed using seven items on a 5-point Likert scale in which score 1 for ‘strongly disagree’, 2 for ‘disagree’, 3 for ‘not sure’, 4 for ‘agree’ and 5 for ‘strongly agree’. The scores ranged from 7–35. The module and questionnaire underwent preliminary testing by 30 nurses in health centers in Kota Bharu to assess their content and face validities. Based on the results of this preliminary testing, the module and questionnaire were revised to address ambiguities, sensitive items, and misinterpretations of information and items.

#### Data collection

The study was explained to the staff nurses in both the intervention and control groups, and written informed consent was obtained. At the baseline, the self-administered questionnaires were distributed. For those in the intervention group, the researchers conducted two training sessions on the counseling module that explained and clarified its content. Each training session lasted about two hours. At the end of each training session, role play was conducted to enhance participants’ understanding of the expectations during counseling sessions. In addition, each participant in the intervention group was given the flipchart and the pamphlet. In contrast, those in the control group were encouraged to self-read and then perform antenatal exercise counseling independently during their routine practices.

Participants in both groups were required to provide antenatal-exercise counseling to at least 10 pregnant women who had no medical or obstetrical problems. Then, at week 4, both groups were administered a similar questionnaire. At that time, each participant in the control group was given the flipchart and pamphlet to help during antenatal-exercise counseling.

#### Data analysis

The data were entered and analysed using SPSS version 20. Before analysis, all data were checked and cleaned. An analysis of variance (ANOVA) and a repeated measure analysis of covariance (ANCOVA) were conducted by controlling for the practice in Maternal and Child Clinic and formal training. Practice in Maternal and Child Clinic refers to the duration of working in Maternal and Child Clinic. Formal training refers to any special courses and workshop on antenatal exercise counseling. It was categorised into ‘yes’ for those had ever attended courses and workshops and ‘no’ for those who never attended any courses or workshops on antenatal exercise counseling. Midwives are referring to nurses who underwent midwifery post basic training.

## Results

The intervention and control groups each contained 66 participants, all of whom responded, meaning that there was a response rate of 100%. Table 1 shows the participants’ baseline characteristics. The intervention and control groups differed significantly in two variables: duration of practice in maternal and child health clinics and formal training received on antenatal-exercise counseling. Therefore, in the subsequent analysis, these variables were adjusted for.

### Knowledge of Antenatal-Exercise Counseling

The ANOVA found no significant difference (*P =* 0.827) in the baseline scores of the intervention and control groups regarding knowledge (Table 2). The repeated measure ANCOVA, after adjusting for known factors affecting knowledge i.e. duration of practice and formal training, found a significant difference (*P* < 0.001) in the knowledge scores of the intervention and control groups at both baseline and at week 4. The estimated marginal mean of the knowledge scores was higher in the intervention group than in the control group.

Model refinement and assessment showed that the residual plots indicated that both the overall model fitness and the equal variance assumption were satisfied. The normality of standardised residuals was appropriate. The variable functional form for duration of practice was appropriate. There were no outliers. Therefore, the above model was considered to be the final model.

### Self-Efficacy on Antenatal-Exercise Counseling

The ANOVA found no significant difference (*P =* 0.089) in the mean baseline self-efficacy scores of the control and intervention groups (Table 3). The repeated measures ANCOVA, after adjusting for known factors affecting self-efficacy i.e. duration of practice and for formal training, found a significant difference (*P =* 0.005) in the self-efficacy scores of the two groups. The estimated marginal mean of the self-efficacy scores was higher in the intervention group than in the control group.

Model refinement and assessment showed that the residual plots indicated that both the overall model fitness and the equal variance assumption were satisfied. The normality of standardised residuals was appropriate. The variable functional form for duration of practice was appropriate. There were no outliers. Therefore, the above model was considered to be the final model.

## Discussion

The present study found that the module produced a significant improvement in nurses’ knowledge of antenatal-exercise counseling. This finding is similar to the results of another educational interventional study on the knowledge and practices of nurses and primary care doctors in Brazil who provide maternity care to pregnant women. The knowledge scores increased among the healthcare providers and the pregnant women who were cared by the intervention group reported receiving better advice on exercise and eating compared to the control group (18). The nurses and primary-care doctors underwent 16 h of training, while in the present study, the nurses underwent a total of 4 h of training with the module to produce a significant different. Both of these studies found that training had positive effects on knowledge levels. Good knowledge and adequate information may help nurses to confidently conduct counseling, and counseling has been shown to effectively sustain physical activity throughout pregnancy (19).

Nurses’ adherence to their training is important to ensuring that accurate information is delivered to antenatal women. In one study, only 19% of the healthcare providers wrote advice or dispensed pamphlets about exercising during pregnancy upon receiving questions from patients (20). Certified nurse midwives were the most likely to provide written instruction (35%), which facilitates understanding of the content of counseling. Indeed, the use of printed materials during brief counseling in primary-care settings was found to promote an active lifestyle (21).

In the present study, the baseline data showed that approximately 81.8% of those in the intervention group and 68.2% of those in the control group knew that the recommended intensity level of exercise during pregnancy was moderate. The remaining participants thought that low-intensity exercise was sufficient for pregnant women. This latter percentage was higher than that in a study in South Africa, in which only 15% of respondents believed that low-intensity exercise was adequate for pregnant mothers. This difference in results was probably due to the different demographic backgrounds of the South African study’s participants, which included doctors, obstetricians, gynecologists and other specialists (20).

Other studies have reported an even higher prevalence of misconception, with 99.5% of obstetricians in one study preferring to recommend low-intensity exercise (22). In another study, providers erroneously regarded antenatal mothers as being at high risk of injury while exercising (9). Still another study underscored a reason why this misconception among healthcare providers needs to be addressed, as it found that Asian women were likely to reduce their activity levels during the third trimester (23).

Regarding the total duration of exercise per week, the majority of participants in the present study agreed that the optimal duration of exercise per week was only 120 min instead of 150 min, indicating a lack of awareness of the recommended duration of moderate-intensity exercise for pregnant women (2, 16). One study found that half the healthcare providers, including obstetricians, midwives, and family-medicine physicians, were unfamiliar with the current recommendations (8).

In the present study, most participants were unaware of the correct point at which to initiate exercise. The Manual on Antenatal and Postnatal Exercise Programme in Health Clinics recommends that pregnant women begin exercising at 16 weeks of gestation (17). Incorrect information might compromise a pregnancy. For example, high-impact exercise early in pregnancy has been associated with an increased risk of miscarriage (24).

In the present study, most participants agreed that those who exercised should continue doing so regularly during pregnancy. Similar findings were reported in studies conducted in Texas (25) and Michigan (22). In addition, the participants in the present study knew that antenatal exercise posed very minimal risk to the foetus. A study has shown a lower frequency of macrosomia in babies born to women who performed exercise regularly and were not associated with intrauterine-growth restrictions (26).

The nurses who used the module significantly improved the self-efficacy of the antenatal-exercise counseling that they provided. Individual counseling has been shown to be a feasible method for healthcare providers to encourage pregnant women to exercise (19). In the present study, a total of 31% of participants in both groups received training on antenatal-exercise counseling, compared to 83% of the participants in another study (8). This difference might be due to the limited number of workshops on antenatal-exercise counseling available in the vicinity of the present study.

Counseling on changing behaviour and lifestyle choices requires a certain approach to ensure its effectiveness. A national survey of nurses, physicians, and physiotherapists in Finland found that they perceived health counseling as difficult (27). Nurses have identified inadequate training as one of the barriers to providing physical-activity counseling in primary care (28). The results of both these studies indicate a strong need for training for healthcare providers. Those who received training were twice as likely to provide counseling (29).

The present study has some limitations. First, it was conducted over a short period, four weeks, which may have compromised the knowledge and self-efficacy outcomes by not reflecting the actual levels of knowledge retained and self-efficacy developed. Second, the majority of participants were Malay; therefore, the findings cannot be generalised to other populations. Third, the questionnaire was assessed for face and content validity only. Construct validity was not evaluated because of time constraints.

## Conclusion

The module significantly increased nurses’ knowledge and self-efficacy regarding delivering antenatal-exercise counseling. All the materials are practical and convenient to use in health centers and therefore, are recommended for use in nurses’ routine counseling sessions to promote healthy lifestyles among pregnant women. Antenatal visits are an opportune time for healthcare providers to promote the importance of an active lifestyle.

## Figures and Tables

**Table 1:** Baseline characteristics of study participants

Variable	Control (*n* = 66)		Intervention (*n* = 66)		*P*-value

	mean (SD a)	*n* (%)	mean (SD a)	*n* (%)	
Age (years)	41.3 (7.0)		41.9 (6.8)		0.651 b
Practice in MCH (years)	7.7 (5.4)		10.5 (6.8)		0.010 b
Races					
Malay		65 (98.5)		64 (97.0)	0.604 c
Non-Malay		1 (1.5)		2 (3.0)	
Midwives					
Yes		64 (97.0)		63 (95.5)	0.648 c
No		2 (3.0)		3 (4.5)	
Formal training					
Yes		8 (12.1)		33 (50.0)	< 0.001^c^
No		58 (87.9)		33 (50.0)	

Notes: MCH = maternal and child health;

a Standard deviation;

b *t*-test;

c Chi-square

**Table 2:** Knowledge scores on antenatal exercise counseling between control and intervention groups

Group	Crude mean at baseline	EMM^b^ (95% CI^c^)		*F* stat^d^ (df^e^)	*P*-value^f^

	(SD^a^)	Baseline	Week-4		
Control	25.0 (3.64)	25.0 (23.56, 28.94)	27.4 (26.52, 28.29)	73.9 (1, 127)	< 0.001
Intervention	24.9 (3.51)	23.8 (22.50, 25.14)	33.9 (33.29, 34.53)		

Notes:

a standard deviation;

b estimated marginal mean;

c confidence interval;

d *F* statistic;

e degree of freedom;

f Repeated measure ANCOVA after adjusting for practice in Maternal and Child Clinic and formal training.

Independence, normality and equal variance assumptions were met

**Table 3:** Self-efficacy scores on antenatal exercise counseling between control and intervention groups

Group	Crude mean at baseline	EMM^b^ (95% CI^c^)		*F* stat^d^ (df^e^)	*P*-value^f^

	(SD^a^)	Baseline	Week-4		
Control	26.2 (3.66)	27.1 (23.73, 28.45)	27.4 (26.03, 28.74)	39.2 (1, 128)	0.005
Intervention	27.4 (4.25)	27.4 (26.33, 28.46)	31.3 (30.55, 32.03)		

Notes:

a standard deviation;

b estimated marginal mean;

c confidence interval;

d *F* statistic;

e degree of freedom;

f Repeated measure ANCOVA after adjusting for practice in Maternal and Child Clinic and formal training.

Independence, normality and equal variance assumptions were met
